# Long noncoding RNA *AFAP1-AS1* acts as a competing endogenous RNA of miR-423-5p to facilitate nasopharyngeal carcinoma metastasis through regulating the Rho/Rac pathway

**DOI:** 10.1186/s13046-018-0918-9

**Published:** 2018-10-16

**Authors:** Yu Lian, Fang Xiong, Liting Yang, Hao Bo, Zhaojian Gong, Yumin Wang, Fang Wei, Yanyan Tang, Xiayu Li, Qianjin Liao, Hui Wang, Ming Zhou, Bo Xiang, Xu Wu, Yong Li, Xiaoling Li, Xiang Chen, Guiyuan Li, Can Guo, Zhaoyang Zeng, Wei Xiong

**Affiliations:** 1 The Key Laboratory of Carcinogenesis of the Chinese Ministry of Health, Xiangya Hospital, Central South University, Changsha, Hunan China; 2 Department of Reproductive medicine, Ganzhou Hospital Affiliated to Nanchang University, NanChang, Jiangxi China; 3 The Key Laboratory of Carcinogenesis and OCancer Invasion of the Chinese Ministry of Education, Cancer Research Institute and School of Basic Medical Science, Central South University, Changsha, Hunan China; 4 Hunan Key Laboratory of Nonresolving Inflammation and Cancer, Disease Genome Research Center, the Third Xiangya Hospital, Central South University, Changsha, Hunan China; 5 Hunan Key Laboratory of Translational Radiation Oncology, Hunan Cancer Hospital and the Affiliated Cancer Hospital of Xiangya School of Medicine, Central South University, Changsha, Hunan China; 6 Department of Chemistry, University of North Dakota, Grand Forks, North Dakota USA; 7 Department of Cancer Biology, Cleveland Clinic, Lerner Research Institute, Cleveland, OH USA

**Keywords:** Long noncoding RNA (lncRNA), *AFAP1-AS1*, miR-423-5p nasopharyngeal carcinoma (NPC), Competing endogenous RNA (ceRNA)

## Abstract

**Background:**

Actin filament-associated protein 1 antisense RNA 1 (*AFAP1-AS1*), a long noncoding RNA, is significantly highly expressed and associated with metastasis and poor prognosis in many cancers, including nasopharyngeal carcinoma (NPC). In this study, we aim to identify the role of *AFAP1-AS1* acting as an oncogenic lncRNA to promote NPC metastasis.

**Methods:**

The role of *AFAP1-AS1*, miR-423-5p, and * FOSL2* in NPC metastasis was investigated in vitro and in vivo. Bioinformatics analysis and luciferase activity assays were used to identify the interaction between *AFAP1-AS1*, miR-423-5p, and *FOSL2*. Additionally, real-time PCR and western blotting were used to assess the function of *AFAP1-AS1* acting as an oncogenic lncRNA to promote NPC progression by regulating miR-423-5p and the downstream Rho/Rac pathway.

**Results:**

In this study, we determined that *AFAP1-AS1* functions as a competing endogenous RNA in NPC to regulate the Rho/Rac pathway through miR-423-5p. These interactions can mediate the expression of *RAB11B*, *LASP1*, and *FOSL2* and accelerate cell migration and invasion via the Rho/Rac signaling pathway or *FOSL2*. *AFAP1-AS1* and *FOSL2* could competitively bind with miR-423-5p to regulate several molecules, including *RAB11B* and *LASP1* of the Rho/Rac signaling pathway. *AFAP1-AS1* can also regulate the expression of *LASP1,* which was transcriptionally regulated by *FOSL2*, resulting in increased migration and invasion of NPC cells via the Rho/Rac signaling pathway.

**Conclusions:**

The observations in this study identify an important role for *AFAP1-AS1* as a competing endogenous RNA (ceRNA) in NPC pathogenesis and indicate that it may serve as a potential target for cancer diagnosis and treatment.

**Electronic supplementary material:**

The online version of this article (10.1186/s13046-018-0918-9) contains supplementary material, which is available to authorized users.

## Background

Nasopharyngeal carcinoma (NPC) is a head and neck epithelial malignancy that occurs frequently in Southern China [1–5]. Epstein-Barr virus exposure [6–15], diet, and genetic factors [16] are implicated in its etiology. Although radiotherapy is an effective treatment for NPC patients in the early stages of the disease [17–20], the majority (75–90%) of NPC cases are predisposed to metastasis at initial diagnosis [21–24], which hampers efficacious treatment and poses a high risk of disease recurrence. Enhancing our understanding of the molecular mechanisms underlying NPC may promote the development of effective metastasis-targeted therapy and improve the overall prognosis of patients with this disease.

Noncoding RNAs, including microRNAs (miRNAs), long noncoding RNAs (lncRNAs) [25–33] and circular RNAs (circRNAs) [34–37], have important roles in the regulation of gene expression and are involved in the development of a variety of human diseases, including cancer. The lncRNA actin filament associated protein 1 antisense RNA 1 (*AFAP1-AS1*) maps to the antisense DNA strand of *AFAP1* and was first identified as highly expressed in esophageal cancer [38]. *AFAP1-AS1* is also highly expressed in lung cancer and pancreas cancer, and hepatocellular carcinoma [39–43]. We previously discovered that *AFAP1-AS1* was significantly highly expressed in NPC and associated with metastasis and poor prognosis [44, 45]. Numerous cytoskeleton proteins, including proteins in the small GTPase Rho/Rac signaling pathway, were identified and significantly regulated using the liquid chromatography-tandem mass spectrometry (LC-MS/MS) by si*AFAP1-AS*, suggesting that *AFAP1-AS1* may regulate NPC migration and invasion through this pathway; however, as a newly identified lncRNA, the mechanism by which *AFAP1-AS1* regulates the Rho/Rac signaling pathway remains unknown.

A new model of lncRNA involvement in gene regulation has recently been proposed, called competing endogenous RNA (ceRNA). This model proposes that lncRNAs and mRNA transcripts affect one another by competitively combining with miRNA response elements (MREs) to influence posttranscriptional regulation [46–48]. In this way, lncRNAs bind with miRNAs to modulate regulation of protein-coding gene expression and participate in the regulation of cell behavior. These interactions between mRNAs, miRNAs, and lncRNAs generate complex regulatory networks [49]. In this study, we found that *AFAP1-AS1* acts as a ceRNA, competitively binding with miR-423-5p and directly regulating *RAB11B* and *LASP1* genes in the Rho/Rac pathway. *AFAP1-AS1* can also regulate the expression of *FOSL2* and its downstream genes by competing with *FOSL2* to bind miR-423-5p, resulting in increased migration and invasion of NPC through the Rho/Rac signaling pathway.

## Methods

### Tissues and cell lines, chemicals, plasmids, and transfection

Thirty-two nasopharyngeal carcinoma samples and 13 nontumor nasopharyngeal epithelium tissues were obtained from patients. All samples were collected with the consent of patients and the experiments were approved by the ethics committee of Hunan Cancer Hospital, Changsha, China. All fresh tissues were immersed in RNALater (Ambion, Austin, TX, USA). The tissue samples were stored in a − 80 °C laboratory freezer. RNA was isolated using TRIzol reagent (Invitrogen, Carlsbad, CA, USA) according to the manufacturer’s protocol. The NPC cell lines 5-8F and HNE2 were maintained in our laboratory in RPMI 1640 medium supplemented with 10% fetal bovine serum (FBS; Invitrogen), penicillin (100 U/ml, Sigma-Aldrich, St. Louis, MO, USA), and streptomycin (100 mg/ml, Sigma-Aldrich). All cell lines had been recently tested for mycoplasma contamination and identified no cross-contaminated cell lines or mycoplasma contamination.

The synthetic miR-423-5p mimics and inhibitors were supplied by Ruibo Co. (Guangzhou, China). siRNAs and nontargeting scrambled control siRNAs were provided by Invitrogen. The sequences of the *AFAP1-AS1*- and *FOSL2*-targeting siRNAs are listed in Additional file 1: Table S1. To construct the *AFAP1-AS1* expression vector, the entire *AFAP1-AS1* sequence was amplified by reverse transcription PCR (RT-PCR) and cloned into the pcDNA3.1 vector. The open reading frame of *FOSL2* (NM_005253) was cloned into the pENTR vector and tagged with the C-terminal Flag and His peptide sequences was purchased from the Vigene Co. (Rockville, MD, USA). Transfection of cells with plasmids, siRNAs, and miRNAs was performed using Lipofectamine 3000 (Life Technologies, Grand Island, NY, USA) or HiPerFect (Qiagen, Hilden, Germany) transfection reagents, according to the manufacturer’s instructions.

### Online expression profiles analysis

NPC data sets and the corresponding clinical data were downloaded from publicly available GEO databases. These included 62 patients with NPC and 6 non-NPC nasopharyngeal epithelial tissues from GSE73460 [50], 6 patients with NPC and 12 non-NPC epithelial tissues from GSE32906 [50] and 122 patients with NPC with survival status from GSE70970 [51].

### Bioinformatics analysis, reporter plasmids, and luciferase activity assays

Five software tools, RNAhybrid, RNA22, PITA, TargetScan, and RegRNA, were used for miRNA target prediction in this study. For miRNA target validation assays, 58 bp fragments of the *AFAP1-AS1* sequence, or the *RAB11B*, *LASP1*, *RAC1*, and *FOSL2* 3′-UTRs containing the wild-type (WT) and mutant (MT) binding sites for miR-423-5p, were cloned into the pMIR-REPORT vector. The sequences of these synthetic oligonucleotides are listed in Additional file 1: Table S1. Cells (5-8F and HNE2) were cotransfected with pMIR-REPORT vectors containing the WT sequences of the *RAB11B*, *RAC1*, *LASP1*, or *FOSL2* 3′-UTRs or their corresponding mutants, along with the pRL-TK control vector and miR-423-5p mimics, inhibitors, or negative control.

For the *LASP1* promoter activity analysis, the Promoter 2.0 Prediction Server (http://www.cbs.dtu.dk/services/Promoter/) was used to predict potential AP1-binding sites in the *LASP1* promoter sequence. Three tandem the wild type or mutant AP1-binding sites were inserted into the TA-luciferase vector as synthetic oligonucleotides encoding the wild type or mutant AP1-binding sites (Additional file 1: Table S1).

The AP-1 cis-element reporter plasmid (the AP-1 reporter), which contains multiple conserved AP1-binding sites for sensitive detection of AP-1 transcriptional activity, was purchased from Promega (pAP1-TA-luc; Promega, Fitchburg, WI, USA) to measure the AP-1 transcriptional activity. Plasmid transfection and luciferase assays were performed as described previously [52]. Cells were harvested 48 h posttransfection and assayed using a Dual Luciferase Assay (Promega) according to the manufacturer’s instructions. All transfection assays were carried out in triplicate.

### Wound healing and transwell assays

For wound healing assays, 5-8F and HNE2 cells were seeded and grown to 90% confluence in 6-well culture plates, and a 200 μl pipet tip was used to create a scratch in the cell monolayer. Images were captured and measured with an ocular ruler to ensure that all wounds were the same width at the beginning of each experiment.

For transwell assays, 5-8F and HNE2 cells in 200 μl serum-free media were placed into upper transwell chambers (8.0 μl pore size, BD Biosciences, Franklin Lakes, NJ, USA) for invasion assays after transfection, according to the manufacturer’s protocol. Invaded cells were fixed and stained with methanol and 0.1% crystal violet, counted under a microscope, and imaged. Independent experiments were carried out in triplicate.

### Western blotting

Proteins were extracted and separated according to previously described methods. Membranes were incubated overnight at 4 °C with primary anti-RHOA, anti-RHOC, anti-RAC2, anti-RAB10, anti-RAB11B, anti-RAB11A, anti-RHOGDI, anti-PFN1, anti-LASP1, or anti-FOSL2 antibodies (Proteintech, Wuhan, China). Blots were visualized by exposure to X-ray films using an enhanced chemiluminescence detection system (EMD Millipore, Burlington, MA, USA). Blots were probed with anti-GAPDH antibody (Cell Signaling Technology, Danvers, MA, USA) as an endogenous control for equal loading.

### Real-time PCR

Total RNA was isolated with TRIzol reagent (Invitrogen) according to the manufacturer’s protocol. For miRNA real-time PCR, cDNA was synthesized using the miScript system (Ruibo) according to the manufacturer’s instructions. miR-423-5p expression levels were measured using a Ruibo miRNA primer assay (Ruibo) and a miDETECT Track miRNA qRT-PCR kit (Ruibo) according to the manufacturer’s instructions. Data were normalized to the small nuclear RNA RNU6B (U6 snRNA) expression level. For mRNAs and lncRNAs, real-time PCR expression analysis was carried out using a SYBR green real-time PCR kit (Applied Biological Materials, Inc., Richmond, BC, Canada). Data were normalized to the *GAPDH* expression level and further normalized to the negative control, unless otherwise indicated. The primers used for PCR are listed in Additional file 1: Table S1. Fold changes in expression were calculated using a relative quantification (2^-ΔΔCt^) method. All reactions were performed in triplicate and repeated in three independent experiments.

### in vivo nude mouse models

Male BALB/C nude mice (5–6 weeks of age, 16–18 g) were maintained in the experimental animal center of Central South University in a pathogen-free facility. The Institutional Animal Ethics Committee of Central South University approved this animal study. Mice were divided into four groups, including the over-AFAP1-AS1, negative control (NC), miR-423-5p, and over-FOSL2 groups. For metastasis assay, 2 × 10^6^ 5-8F cells in a volume of 200 ml with corresponding treatment were tail-vein injected into five-week-old male BALB/c nude mice which were randomly divided into four groups (*n* = 10 for each group). 60 days later, the mice were killed, and all the lungs are surgically removed and the number of macroscopically visible pulmonary metastases nodules per mouse was counted by two experienced pathologists. Then, the lung tissues were fixed in 10% neutral phosphate-buffered formalin, followed by HE staining.

### Statistical analysis

All continuous variables are presented as the means ± standard deviation (SD), and categorical data are expressed as percentages. GraphPad Prism 5.0 (GraphPad Software, Inc., La Jolla, CA, USA) was used for statistical analysis. Student’s t-test was performed to assess significant differences between two groups using *p* < 0.05 as the significance threshold. Survival analysis was performed by Kaplan-Meier curves and log-rank test for significance. A two-sided *p*-value of < 0.05 was considered statistically significant.

## Results

### Overexpression of *AFAP1-AS1* accelerates NPC cell migration and invasion

**Fig. 1:**
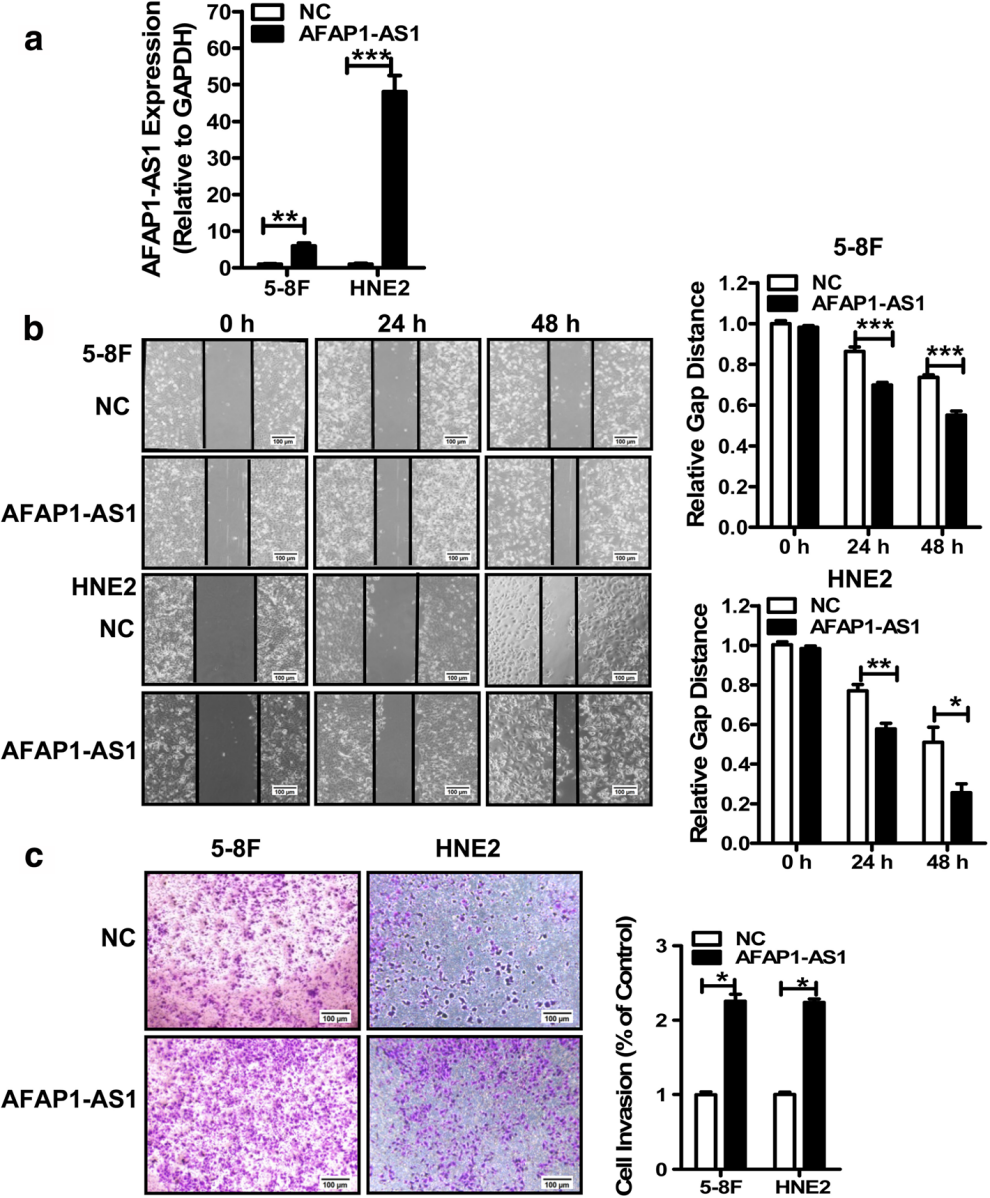
Overexpression of *AFAP1-AS1* stimulates NPC cells migration and invasion of in vitro. **a**. *AFAP1-AS1* levels were detected by qRT-PCR in 5-8F and HNE2 cells after transfection with the pcDNA3.1-*AFAP1-AS1* (*AFAP1-AS1*) vector and the empty pcDNA3.1 vector (NC). **b**. Wound healing assays using 5-8F and HNE2 cells transfected with the pcDNA3.1-*AFAP1-AS1* (AFAP1-AS1) or the pcDNA3.1 (NC) vectors. The relative ratio of wound closure per field is shown on the right. Scale bars, 100 μm. *, *p* < 0.05; **, *p* < 0.01; ***, *p* < 0.001. **c**. Transwell analysis of 5-8F and HNE2 cells transfected with the pcDNA3.1-*AFAP1-AS1* (AFAP1-AS1) or the pcDNA3.1 vectors (NC). The relative ratio of invasive cells per field is shown on the right. For all quantitative results, data are presented as the means and standard errors of the mean (± SEM) from three independent experiments. Scale bars, 100 μm. *, *p* < 0.05; **, *p* < 0.01; ***, *p* < 0.001

We first confirmed the function of *AFAP1-AS1* in NPC cells migration and invasion. The overexpressing *AFAP1-AS1* vector was constructed and transfected into the NPC cell lines 5-8F and HNE2, and the expression of *AFAP1-AS1* was confirmed by real-time PCR (Fig. 1a). Wound healing and transwell assays demonstrated that overexpression of *AFAP1-AS1* accelerated 5-8F and HNE2 cell migration (Fig. 1b) and invasion (Fig. 1c).

### *AFAP1-AS1* physically interacts with miR-423-5p in NPC cells

**Fig. 2:**
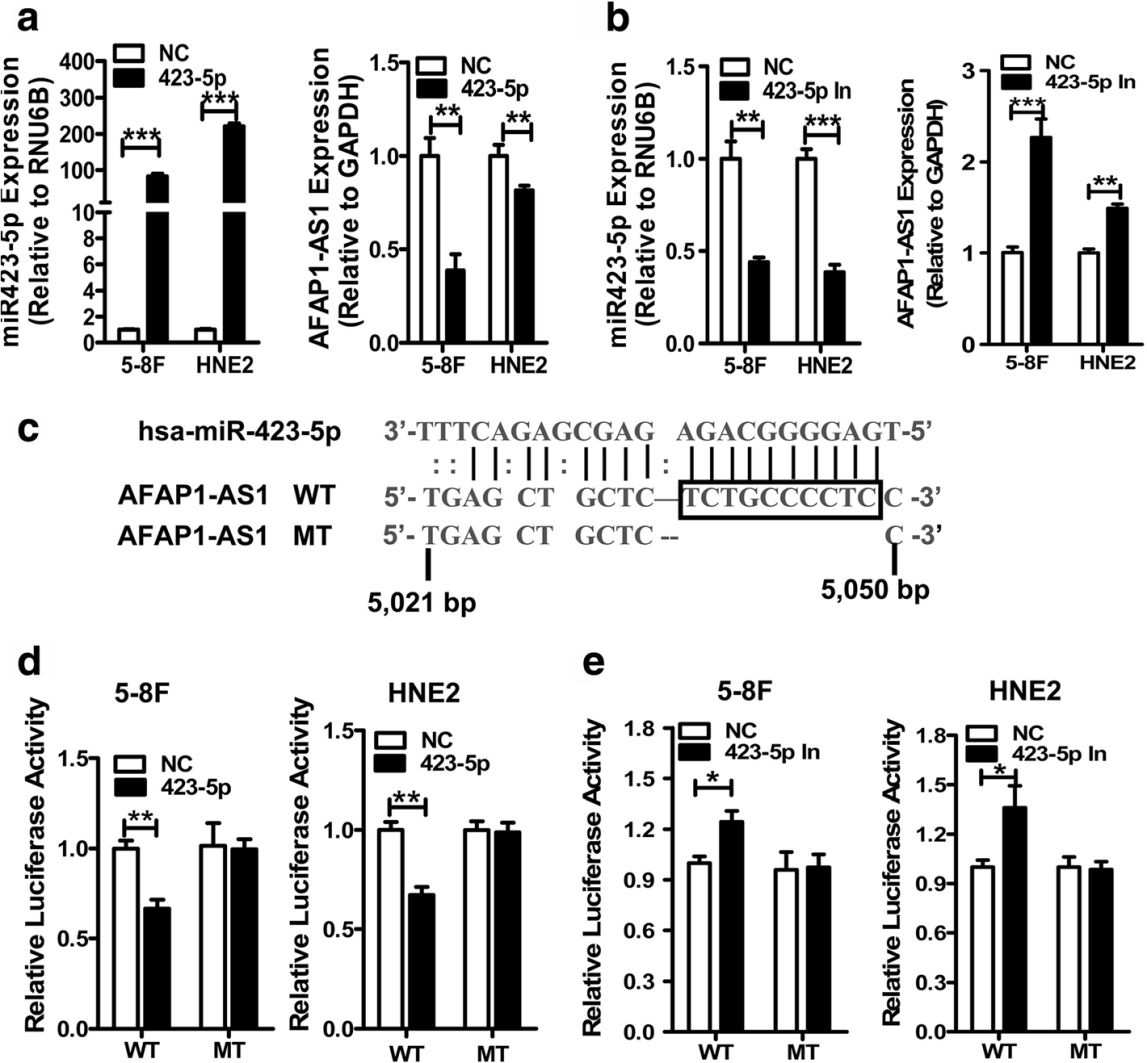
miR-423-5p could bind with the *AFAP1-AS1* sequence. **a**. The expression level of *AFAP1-AS1* was reduced by miR-423-5p mimics compared with negative controls (NC) in NPC cells transfected with miR-423-5p mimics or negative controls using the qRT-PCR method. **b**. The expression level of *AFAP1-AS1* was induced by miR-423-5p inhibitors (423-5p In) in 5-8F and HNE2 cells transfected with miR-423-5p mimics or negative controls. **c**. Sequence alignment of human miR-423-5p within the *AFAP1-AS1* sequence. The seed sequence of miR-423-5p matches the *AFAP1-AS1* sequence. Mutations within the *AFAP1-AS1* sequence. *AFAP1-AS1* in the mutant luciferase reporter constructs are as shown. **d**. The pMIR-reporter-AFAP1-AS1 vector was used for the luciferase assays. 5-8F and HNE2 cells were transfected with miR-423-5p mimics or control and luciferase reporters containing the wide type or mutant *AFAP1-AS1* sequence, as indicated. Firefly luciferase activity was normalized with Renilla luciferase activity. Relative luciferase activities are presented. Data represent three independent experiments in triplicate. **e**. Luciferase activity was measured in 5-8F and HNE2 cells cotransfected with miR-423-5p inhibitors, negative control, and the luciferase reporter constructs containing the wide type or mutant A*FAP1-AS1* sequence, as indicated. *, *p* < 0.05; **, *p* < 0.01; ***, *p* < 0.001

To identify the mechanism of *AFAP1-AS1* in NPC, we used four programs (PITA, RNAhybrid, RNA22, and RNAreg2.0) to predict potential lncRNA-miRNA interactions involving *AFAP1-AS1*. Among the results, miR-423-5p was selected for further investigation, since it was the only interacting miRNA proposed by all these four tools. Then, whether endogenous *AFAP1-AS1* levels were affected by miR-423-5p was examined through transfection of miR-423-5p mimics or inhibitors into 5-8F and HNE2. Real-time PCR indicated that *AFAP1-AS1* expression was reduced after transfection with miR-423-5p mimics (Fig. 2a) and induced after transfection with miR-423-5p inhibitors in 5-8F and HNE2 cells (Fig. 2b). These results indicated that *AFAP1-AS1* was negatively regulated by miR-423-5p in NPC cells. A total of 2 binding sites for miR-423-5p were predicted in the *AFAP1-AS1* sequence. One binding site between 5021 and 5043 bp of *AFAP1-AS1* (NR_026892, Fig. 2c) with the lowest minimum free energy was selected, and its wild-type (WT) and mutant (MT) sequences were cloned into a pMIR-REPORT luciferase vector for luciferase activity assay. The result showed that transfection of miR-423-5p mimics into 5-8F and HNE2 cells reduced the luciferase activity generated by the wild-type *AFAP1-AS1* reporter (WT), but not that of the empty vector or the reporter vector containing the mutant miR-423-5p binding site (Fig. 2d). Moreover, transfection with miR-423-5p inhibitors increased the luciferase activity of the wild type *AFAP1-AS1* reporter (WT, Fig. 2e), suggesting that miR-423-5p binds with *AFAP1-AS1* through this site.

### miR-423-5p is expressed at low levels in NPC and suppresses cell migration and invasion

**Fig. 3:**
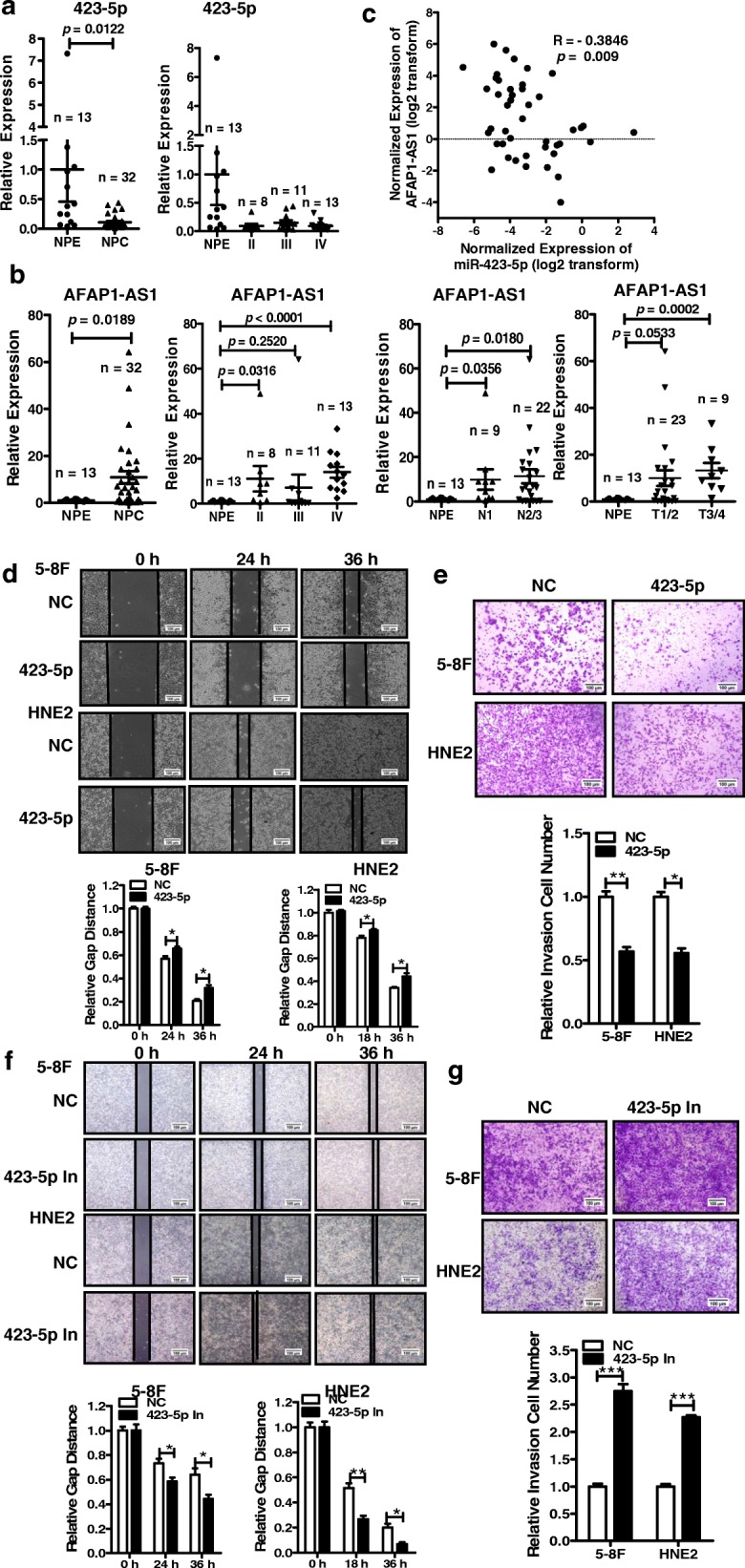
The expression of miR-423-5p was negatively correlated with *AFAP1-AS1* in NPC biopsies and inhibited NPC cell migration and invasion in vitro. **a**. miR-423-5p was lowly expressed in 32 NPC tissues compared with 13 nontumor nasopharyngeal epithelial (NPE) tissues and correlated with the TNM stages of NPC patients as indicated. **b**. *AFAP1-AS1* was highly expressed in the same 32 NPC tissues compared with 13 nontumor tissues and tightly correlated with the TNM stages, lymphoma metastasis, and the T stage of NPC patients, as indicated. **c**. The expression of miR-423-5p was tightly negatively correlated with that of *AFAP1-AS1* in the same 32 NPC tissues and 13 nontumor tissues as indicated. **d**. The invasion ability was measured in 5-8F and HNE2 cells after treatment with miR-423-5p mimics or negative control using wound healing assays. The relative ratio of wound closure per field is shown at the bottom. Scale bars, 100 μm. **e**. Transwell assays were performed to measure the migration ability of miR-423-5p mimics in 5-8F and HNE2 cells after treatment with miR-423-5p mimics or negative control. The relative ratio of invasive cells per field is shown at the bottom. Scale bars, 100 μm. **f**. Wound healing assays of 5-8F and HNE2 cells after treatment with miR-423-5p inhibitors or negative control. The relative ratio of wound closure per field is shown to the bottom. Scale bars, 100 μm. **g**. Transwell assays were performed in 5-8F and HNE2 cells after treatment with miR-423-5p inhibitors or negative control. The relative ratio of invasive cells per field is shown on the bottom. Scale bars, 100 μm. *, *p* < 0.05; **, *p* < 0.01; ***, *p* < 0.001

Based on the above findings, we screened online published miRNA datasets and found that miR-423-5p expression was low-expressed in NPC clinical samples compared with nontumor nasopharyngeal epithelium tissues in a gene expression profiling (GEP) dataset, GSE73460 (Additional file 2: Figure S1a), and another NPC GEP dataset, GSE32906 showed that the expression of miR-423-5p was tightly associated with the TNM stages of NPC patients (Additional file 2: Figure S1b). In the GSE70970 dataset, decreased miR-423-5p expression was also associated with poor overall survival and poor relapse-free survival (Additional file 2: Figure S1c) of NPC patients. Then, the expression of *AFAP1-AS1* and miR-423-5p were examined in 32 NPC clinical samples and 13 nontumor nasopharyngeal epithelium biopsies by qRT-PCR. The results showed that miR-423-5p was lowly expressed in NPC tissues compared with that in nontumor nasopharyngeal epithelium tissues (Fig. 3a). Conversely, *AFAP1-AS1* was highly expressed in the NPC tissues and tightly associated with the clinical TNM stages, neck lymph node metastasis, and the T stages of the patients (Fig. 3b). There was an inverse correlation between *AFAP1-AS1* and miR-423-5p in the NPC tissues (Fig. 3c).

Next, we measured the cell migration and invasion capacity of miR-423-5p in NPC cells. Wound healing and transwell assays demonstrated that miR-423-5p overexpression using mimics inhibited cell migration and invasion compared with those of negative control (Fig. 3d and e), consistently with the functional effects of *AFAP1-AS1* knockdown. Moreover, transfection with miR-423-5p inhibitors had a consequence similar to the function of *AFAP1-AS1* overexpression in 5-8F and HNE2 cells (Fig. 3f and g). Taken together, these results indicated that *AFAP1-AS1* might promote NPC cell migration and invasion via mutually regulated with miR-423-5p.

### *AFAP1-AS1* regulates the Rho/Rac signaling through miR-423-5p

We previously used a proteomics strategy to determine that *AFAP1-AS1* regulates the expression of several small GTPase family members, and modulates cancer cell metastasis via regulation of actin filament integrity [44]. However, the mechanism by which *AFAP1-AS1* regulates the GTPase family members expression remains unknown. Since *AFAP1-AS1* interacts with miR-423-5p, we hypothesized that *AFAP1-AS1* might act as a ceRNA to regulate the GTPase family members through miR-423-5p.

**Fig. 4:**
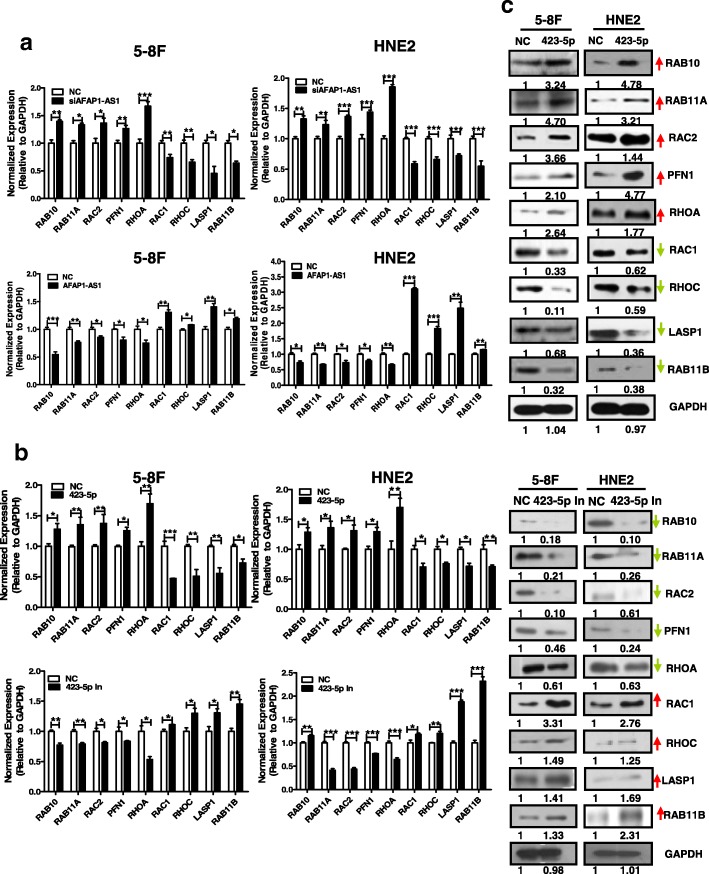
miR-423-5p regulates the small GTPase Rho/Rac signaling pathway through *AFAP1-AS1.* **a**. Many molecules in the Rho/Rac signaling pathway, including *RAB10*, *RAB11A*, *RAC2*, *PFN1*, *RHOA*, *RAC1*, *RHOC*, *LASP1* and *RAB11B*, were regulated at the mRNA level in 5-8F and HNE2 cells by *AFAP1-AS1* overexpression or knockdown. **b**. Many molecules in the Rho/Rac signaling pathway, including *RAB10*, *RAB11A*, *RAC2*, *PFN1*, *RHOA*, *RAC1*, *RHOC*, *LASP1* and *RAB11B*, were regulated at the mRNA level in 5-8F and HNE2 cells by overexpression or knockdown of miR-423-5p. **c**. Expression of Rho/Rac proteins, including *RAB10*, *RAB11A*, *RAC2*, *PFN1*, *RHOA*, *RAC1*, *RHOC*, *LASP1* and *RAB11B*, was examined at the protein level in 5-8F and HNE2 cells after overexpression or knockdown of miR-423-5p.*, *p* < 0.05; **, *p* < 0.01; ***, *p* < 0.001

We first confirmed that *AFAP1-AS1* regulates the small GTPase family members at the mRNA level. After *AFAP1-AS1* knockdown (siRNA) or overexpression in 5-8F and HNE2 cells, multiple members of the Rho/Rac pathway were affected at the mRNA level, as determined by real-time PCR (Fig. 4a), and the expression of these molecules was also altered at the protein level, as revealed by western blotting (Additional file 2: Figure S2); these results were consistent with those of our previous proteomics analysis [44]. Similar results were obtained in 5-8F and HNE2 cells after transfection with miR-423-5p mimics and inhibitors (Fig. 4b and c). miR-423-5p regulated the expression of numerous molecules, including RAB10, RAB11A, RAC2, PFN1, RHOA, RAC1, RHOC, LASP1, and RAB11B, in the Rho/Rac signaling pathway at both the mRNA (Fig. 4b) and protein levels (Fig. 4c), suggesting that *AFAP1-AS1* may regulate the Rho/Rac signaling via miR-423-5p.

### miR-423-5p regulates the Rho/Rac signaling by directly targeting the 3′-UTRs of *RAB11B* and *LASP1*, but not that of *RAC1*

**Fig. 5:**
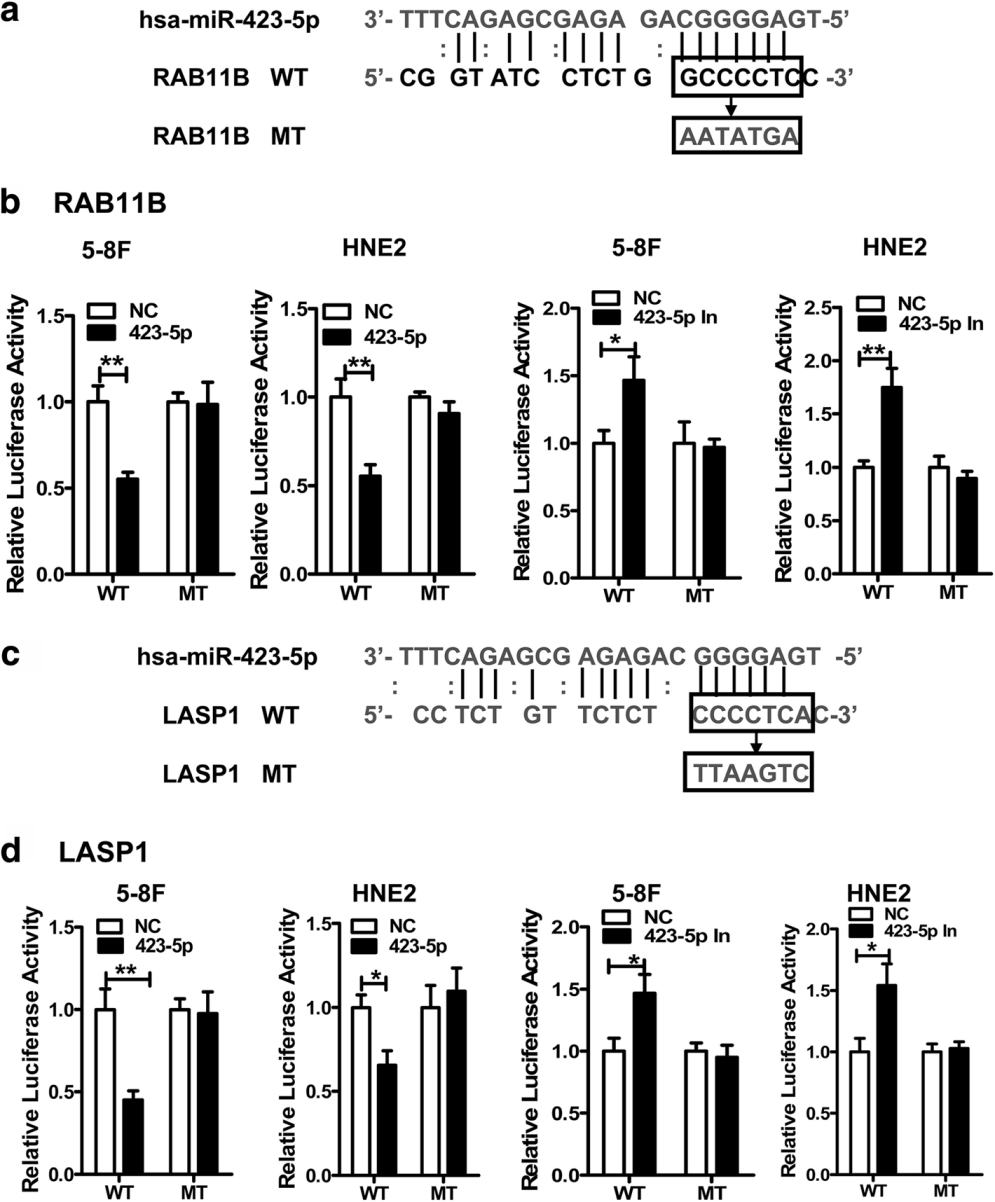
miR-423-5p directly targets the 3′-UTRs of *RAB11B* and *LASP1.* **a** and **c**. Schematic diagrams of miR-423-5p binding sites in the 3′-UTRs of the *RAB11B* (**a**) and *LASP1* (**c**) mRNAs predicted using RNAhybrid software. **b** and **d**. Luciferase activity was measured in 5-8F and HNE2 cells after cotransfection with miR-423-5p mimics or inhibitors and luciferase reporter constructs containing either wild-type or mutated miR-423-5p binding sites from the *RAB11B* (**b**) or *LASP1* (**d**) 3′-UTRs. miR-423-5p mimics attenuated luciferase activity from RAB11B-WT and LASP1-WT constructs, but not from RAB11B-mutant and LASP1-mutant constructs. *, *p* < 0.05; **, *p* < 0.01; ***, *p* < 0.001

As shown in Fig. 4, we found that the expression of RAC1, RHOC, LASP1, and RAB11B was reduced by miR-423-5p mimics and induced by miR-423-5p inhibitors at both the mRNA and protein levels. We next investigated whether miR-423-5p directly targeted these molecules to regulate the Rho/Rac signaling pathway. First, the 3′-UTR sequences of these genes were analyzed using the TargetScan software. Interestingly, the 3′-UTRs of *RAC1*, *LASP1*, and *RAB11B* contained numerous elements exactly complementary to the miR-423-5p seed region. Next, the vectors containing the wild type or mutant (mutations targeting the seed miR-423-5p sequence) *RAB11B*, *LASP1*, and *RAC1* 3′-UTR sequences were constructed, and luciferase analysis showed that miR-423-5p mimics reduced luciferase activity in 5-8F and HNE2 when transfected with vectors containing the wild type *RAB11B* (Fig. 5a and b) and *LASP1* (Fig. 5c and d) 3′-UTR sequences, but not in 5-8F and HNE2 transfected with the wild type *RAC1* 3′-UTR (pMIR-WT) (Additional file 2: Figure S3). The effect of miR-423-5p mimics on the luciferase activity of the vectors containing the mutant *RAB11B* and *LASP1* sequence was abolished in 5-8F and HNE2 cells (Fig. 5). Transfection of miR-423-5p inhibitors in these cells had the opposite effect on the luciferase activity of the *RAB11B* and *LASP1* 3′-UTR reporter constructs (Fig. 5). These results suggested that miR-423-5p regulated the Rho/Rac signaling through direct binding the 3′-UTRs of *RAB11B* and *LASP1*.

### *AFAP1-AS1* acts AS a ceRNA regulating *FOSL2* expression in NPC

**Fig. 6:**
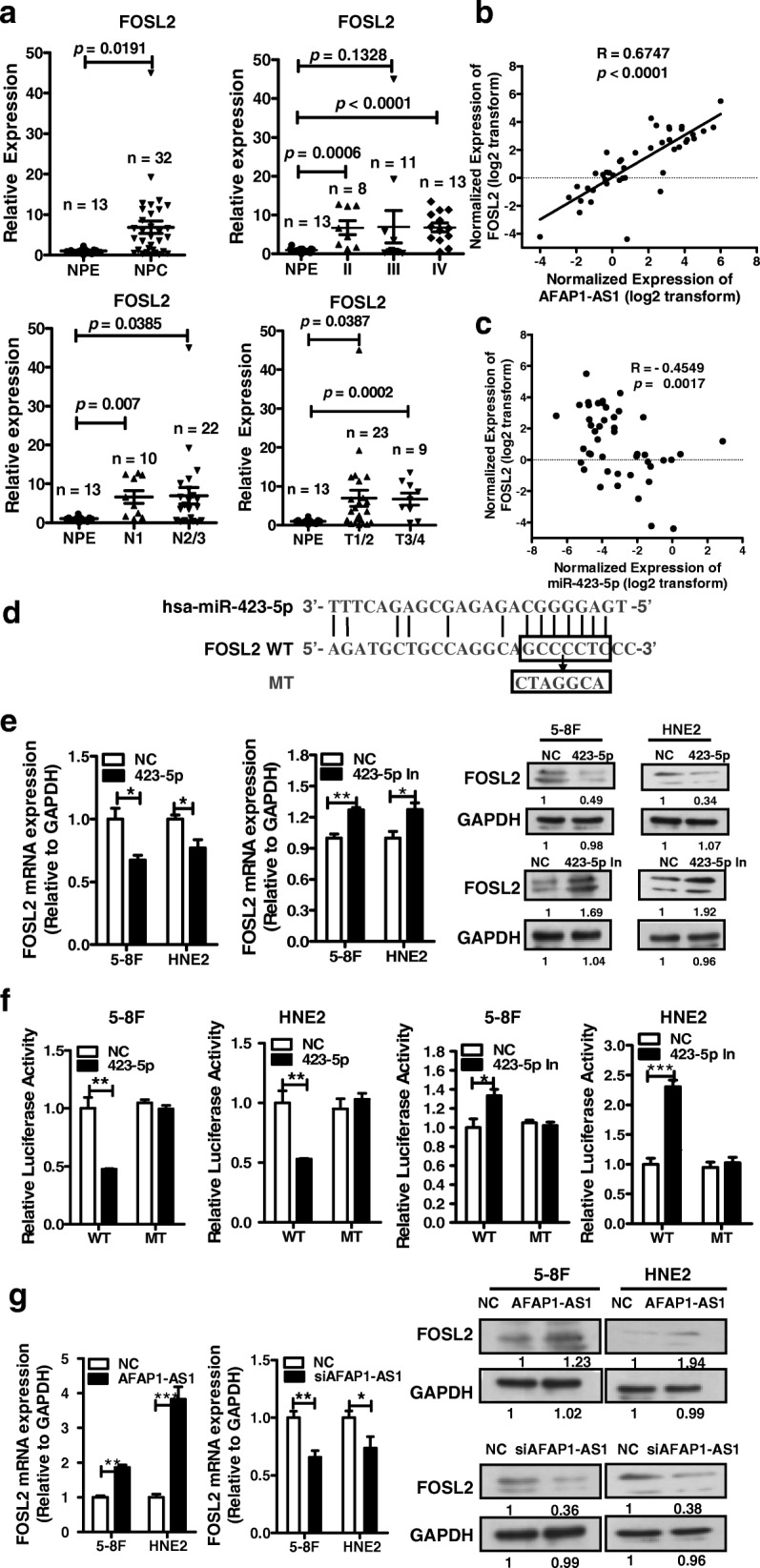
*AFAP1-AS1* acts as a ceRNA to regulate *FOSL2* gene expression through miR-423-5p. **a**. The expression of *FOSL2* and its correlation with the TNM stages, lymphoma metastasis, and T stages in the same 32 NPC tissues and 13 nontumor tissues as indicated. **b**. The expression of *FOSL2* was tightly positively correlated with that of *AFAP1-AS1* in 32 NPC tissues and 13 nontumor tissues as indicated. **c**. The expression of *FOSL2* was tightly negatively correlated with that of miR-423-5p in 32 NPC tissues and 13 nontumor tissues as indicated. **d**. Prediction of miR-423-5p binding sites in the *FOSL2* transcript. The red nucleotides are complementary to the miR-423-5p seed sequences. **e**. The expression of *FOSL2* was regulated by miR-423-5p at the mRNA and protein levels after transfection of miR-423-5p mimics, miR-423-5p inhibitors, or negative control in 5-8F and HNE2 cells. **f**. Luciferase activity was measured in 5-8F and HNE2 cells cotransfected with miR-423-5p inhibitors, miR-423-5p mimics, or negative control, and luciferase reporters containing WT or MT *FOSL2* sequences, as indicated. **g**. The expression of *FOSL2* was examined at the mRNA and protein levels in 5-8F and HNE2 cells after transfection with *AFAP1-AS1* overexpression vector, or *siAFAP1-AS1*

The ceRNA hypothesis posits that specific lncRNA molecules function as sinks for pools of active miRNAs, functionally liberating mRNA transcripts targeted by certain miRNAs. Therefore, the expression of lncRNAs that act as ceRNAs is usually positively correlated with the expression levels of their affected mRNAs. To determine which mRNAs are regulated by *AFAP1-AS1* through miR-423-5p via ceRNA, we first performed bioinformatics analysis to predict targets of miR-423-5p. The NPC gene expression omnibus (GEO) datasets were screened to identify mRNAs whose expression levels were positively correlated with those of *AFAP1-AS1*. The expression of *FOSL2* was positively correlated with that of *AFAP1-AS1* [53] (Additional file 2: Figure S4). Real-time PCR showed that *FOSL2* was also overexpressed in those 32 NPC clinical samples compared with nontumor nasopharyngeal epithelial samples and tightly positively associated with the TNM stages, the T stages, and lymphoma metastasis in patients with NPC (Fig. 6a), positively correlated with the *AFAP1-AS1* expression (Fig. 6b) while negatively correlated with miR-423-5p (Fig. 6c). The 3′-UTRs of *FOSL2* were also predicted with several potential binding sites complementary to the miR-423-5p seed region (Fig. 6d). Transfection of miR-423-5p mimics or inhibitors into 5-8F and HNE2 cells reduced or induced the mRNA and protein levels of *FOSL2*, respectively (Fig. 6e). Luciferase assays also showed that miR-423-5p mimics decreased the luciferase activity of the wild type *FOSL2* 3′-UTR sequence (pMIR-WT), while no effect was observed on the luciferase activity from vectors containing the mutant miR-423-5p binding sites in 5-8F and HNE2 cells. Transfection with miR-423-5p inhibitors had the opposite effect on the luciferase activity generated from the *FOSL2* 3′-UTR (Fig. 6f).

We hypothesized that, as a ceRNA, *AFAP1-AS1* competed with *FOSL2* to bind miR-423-5p; therefore, we determined whether there was specific interplay among *AFAP1-AS1*, miR-423-5p, and *FOSL2*. We monitored both the mRNA and protein levels of *FOSL2* after *AFAP1-AS1* overexpression and knockdown in 5-8F and HNE2 cells. As expected, *AFAP1-AS1* overexpression increased both the mRNA and protein levels of *FOSL2*, and siAFAP1-AS1 transfection reduced *FOSL2* expression (Fig. 6g). Overall, these results suggested that the role of *AFAP1-AS1* in modulating *FOSL2* expression is mediated by its competitive binding with miR-423-5p in 5-8F and HNE2 cells. Together, these data indicated that *AFAP1-AS1* functions as a ceRNA by competitively binding miR-423-5p, thereby relieving the suppression of *FOSL2* expression by miR-423-5p in NPC.

### *FOSL2* accelerates NPC cell migration and invasion

**Fig. 7:**
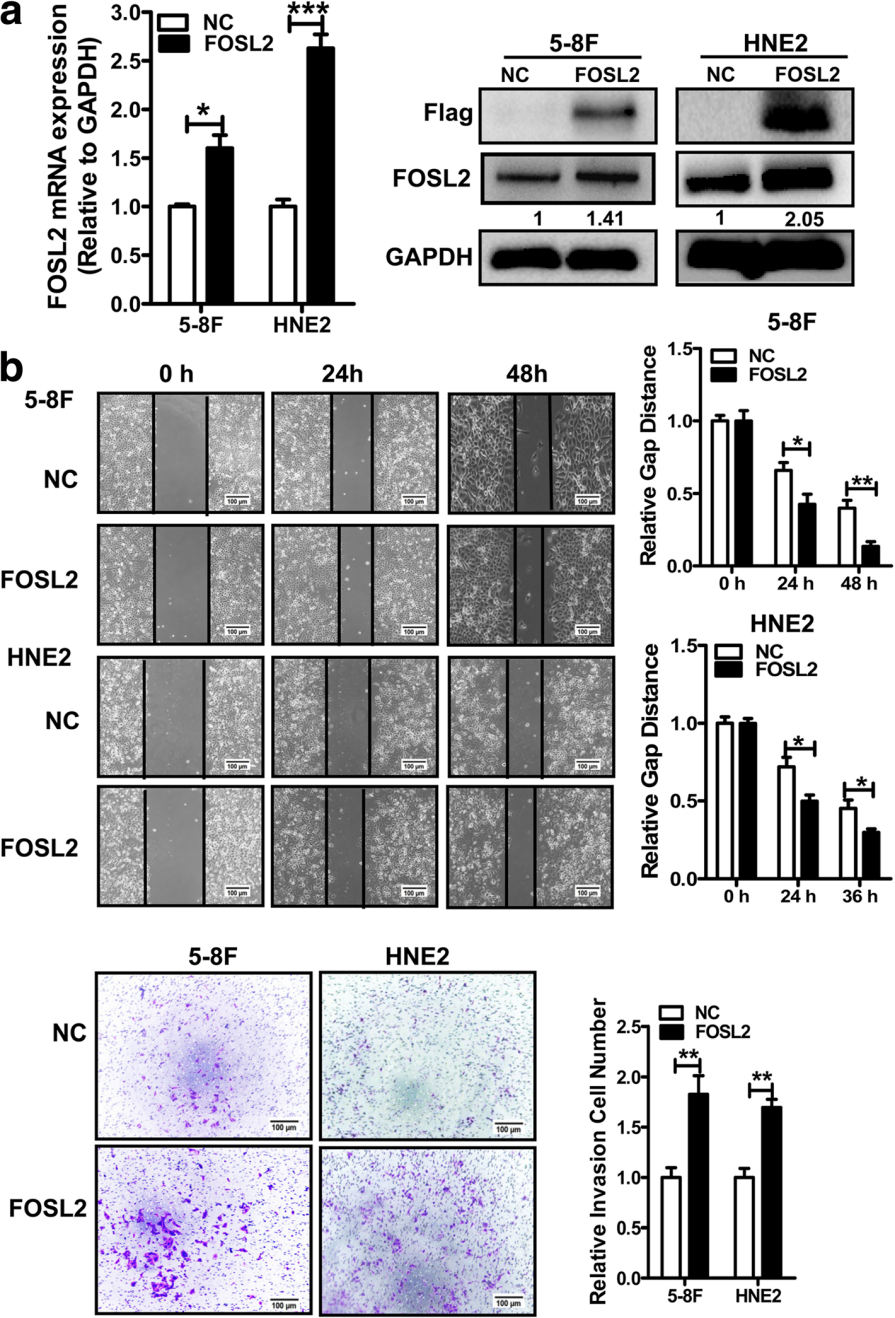
Functional analysis of *FOSL2* in NPC cells. **a**. *FOSL2* mRNA and protein levels were detected in 5-8F and HNE2 cells after transfection with the *FOSL2* overexpression vector (FOSL2) or the empty vector (NC) by qRT-PCR and western blotting. **b**. Wound healing and transwell assays of 5-8F and HNE2 cells were conducted after transfection with pENTR-*FOSL2* or pENTR empty vector. The relative ratio of wound closure per field is shown on the right. Scale bars, 100 μm. **c**. The expression of *FOSL2* was examined at the mRNA and protein levels by qRT-PCR and western blotting in 5-8F and HNE2 cells after transfection with *siFOSL2* or scrambled negative control siRNA. **d**. Wound healing and transwell assays were performed in 5-8F and HNE2 cells after treatment with *siFOSL2* or scrambled siRNA control. The relative ratio of invasive cells per field is shown on the right. Scale bars, 100 μm. *, *p* < 0.05; **, *p* < 0.01; ***, *p* < 0.001

To investigate the function of *FOSL2* in NPC, a *FOSL2* overexpression vector, including three Flag tags, was constructed and its expression confirmed in 5-8F and HNE2 cells (Fig. 7a). Wound healing and transwell assays demonstrated that overexpression of *FOSL2* accelerated cell migration and invasion compared to those in negative control (Fig. 7b). Moreover, *FOSL2* knockdown by siRNA (Fig. 7c) had the opposite effect (Fig. 7d). Taken together, these results indicated that *FOSL2* induces NPC cell migration and invasion.

### *FOSL2* activates transcription of *LASP1* by binding its promoter

**Fig. 8:**
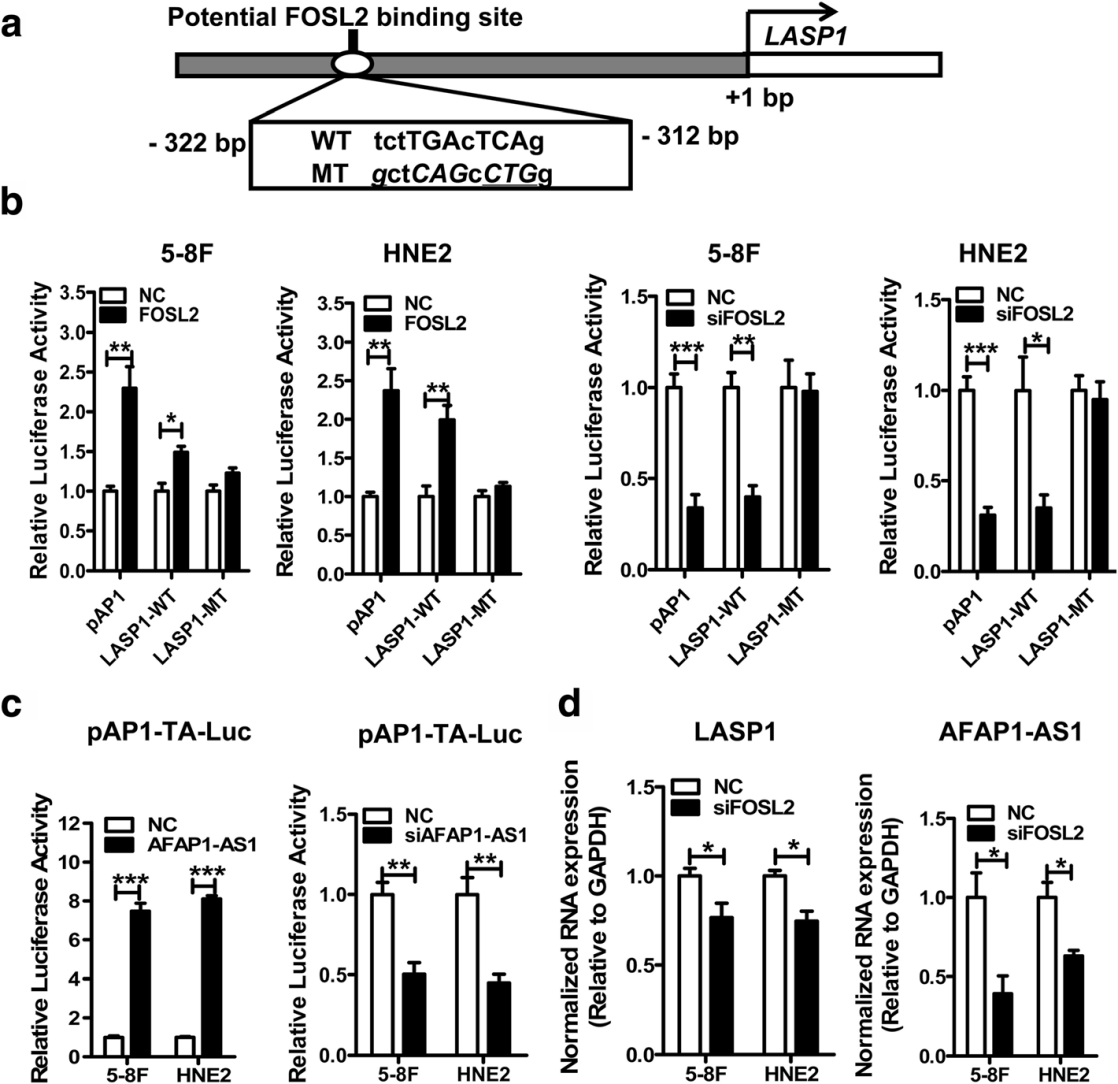
*FOSL2* modulates the transcription of *LASP1* through binding its promoter. **a**. Prediction of potential FOSL2 binding sites in the *LASP1* promoter. The nucleotides that are consensus FOSL2 binding sequences (WT) and mutated sequences (MT) are indicated. **b**. The luciferase activity of the *LASP1* promoter and the AP-1 transcription factor was measured in 5-8F and HNE2 cells cotransfected with the *FOSL2* overexpression vector or *siFOSL2* and luciferase reporters containing the AP-1 luciferase plasmid (pAP1), or the WT or MT *LASP1* promoter, as indicated. Overexpression of *FOSL2* significantly induced the *LASP1* and AP-1 transcription activity. *p < 0.05, **p < 0.01, compared with normal control (NC) group. **c**. The AP-1 transcription activity was examined through transfection of the *AFAP1-AS1* overexpression vector or *siAFAP1-AS1* and the AP-1 reporter plasmid containing the AP-1 consensus sequence. AP-1 transcriptional activity was induced through overexpressing *AFAP1-AS1* or reduced after knockdown of *AFAP1-AS1*. **d**. The expression of *LASP1* and *AFAP1-AS1* was regulated by *FOSL2* after knockdown or overexpression of *FOSL2*

*FOSL2* is a member of the Fos family of transcription factors that heterodimerize with the Jun family members to form the AP-1 complex, which is able to activate or suppress the expression of many genes involved in tumor growth and progression [54]. As a transcription factor, *FOSL2* could modulate the expression of genes in the Rho/Rac pathway. Therefore, we determined whether *FOSL2* could transcriptionally activate downstream genes in the Rho/Rac signaling pathway. Bioinformatics analysis indicated a potential *FOSL2* binding site in the *LASP1* promoter positioned at the − 322 to − 312 bp upstream of the transcription start site (Fig. 8a). Next, luciferase reporter constructs containing the wild-type and mutant *LASP1* promoter sequences, including the potential *FOSL2* binding site, were constructed (Fig. 8a). An AP-1 luciferase vector, containing multiple conserved AP1-binding sites for sensitive detection of the AP1 transcriptional activity, was used as a positive control. As shown in Fig. 8b, *FOSL2* activated transcription from only the wild-type, but not the mutated *LASP1* promoter in these two cell lines. *AFAP1-AS1* also participated in regulation of the transcription activity of the AP-1 complex containing *FOSL2*. Overexpression of *AFAP1-AS1* dramatically increased and knockdown decreased the AP-1 transcriptional activity from a synthetic promoter including the AP-1 consensus-binding site (pAP1-TA-luc) (Fig. 8c). *FOSL2* knockdown also reduced *LASP1* and *AFAP1-AS1* expression (Fig. 8d). These results suggest that *AFAP1-AS1* may regulate the Rho/Rac signaling pathway via the AP-1 transcriptional complex through modulating *FOSL2* activity by competition with miR-423-5p.

### The function of *AFAP1-AS1*, miR-423-5p, and *FOSL2* in NPC metastasis in vivo

**Fig. 9:**
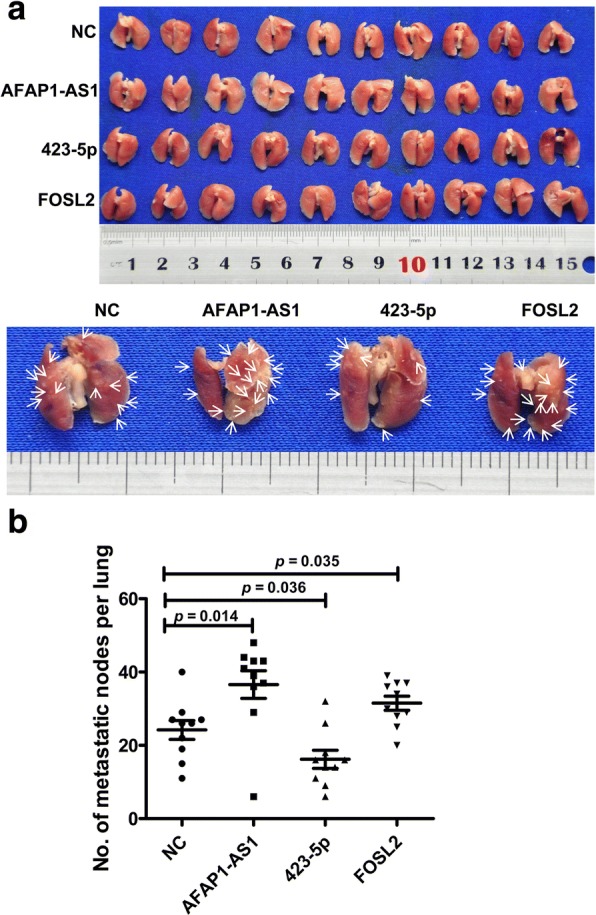
The function of *AFAP1-AS1*, 423-5p, and *FOSL2* in the metastasis of nasopharyngeal carcinoma 5-8F cells in vivo. **a**. Forty nude mice were randomly divided into 4 groups with 10 mice per group. Three experimental groups were injected with 5-8F cells transfected with the *AFAP1-AS1* overexpression plasmid, miR-423-5p mimics, or the *FOSL2* overexpression plasmid through the tail veins, and the control group was injected with 5-8F mock cells (NC). After 8 weeks, the nude mice were sacrificed, and the metastatic tumors were observed (upper panel). Representative lung tissue and metastatic nodes are indicated by arrows (lower panel). **b**. Metastatic nodes per lung in each group

The function of *AFAP1-AS1*, miR-423-5p, and *FOSL2* in metastasis was confirmed in nude mice. *AFAP1-AS1*-, miR-423-5p-, and *FOSL2*-overexpressing 5-8F cell lines were used in this study. The cells were injected into the tail veins of nude mice; 5-8F-mock served as the control. After 60 days, the mice were sacrificed, and the lungs, livers, and lymph nodes were observed. Compared with the control, metastasis to the lungs of 5-8F cells transfected with *AFAP1-AS1* and *FOSL2* significantly increased, while metastasis of 5-8F transfected cells with miR-423-5p mimics were reduced (Fig. 9). These results indicated that *AFAP1-AS1* and *FOSL2* enhanced NPC metastasis and miR-423-5p reduced its metastasis.

## Discussion

A ceRNA hypothesis was proposed by Salmena et al. in 2011 [55]. The theory explains why mRNA, pseudogenes, lncRNA, and other molecules share miRNA response elements (MREs) and bind to identical miRNAs competitively to affect cell status [56]. miRNAs and MREs are two key elements in this hypothesis; miRNAs bind not only to MREs in mRNAs but also to pseudogenes and lncRNAs, influencing mRNA and lncRNA expression levels posttranscriptionally. For example, the tumor suppressor gene phosphatase and tensin homolog (*PTEN*), a negative regulator of the oncogenic phosphoinositide 3-kinase/Akt signaling pathway, provides the best evidence for disease-associated ceRNAs [57]. *PTEN* pseudogene 1 (*PTENP1*) can increase PTEN expression by binding to several miRNAs to suppress proliferation of cancer cells [58, 59]. Conversely, when *PTENP1* is downregulated, more miRNAs are available to inhibit *PTEN* expression, facilitating tumor growth. Both *PTEN* and *PTENP1* are controlled by ceRNA circuitry in many cancers [60]. LncRNAs interact with miRNAs and mRNAs as components of the cellular RNA language, which comprises a complex, finely controlled network.

*AFAP1-AS1* is highly expressed and tightly associated with tumor progression in many cancers including NPC [43]. We previously showed that *AFAP1-AS1* was associated with poor prognosis in NPC and it promoted cell migration and invasion via regulation of actin filament integrity and the Rho/Rac signaling [44]. In this study, we explored the detailed molecular mechanism of *AFAP1-AS1* as a ceRNA in regulation of NPC cell migration and invasion. We hypothesized that *AFAP1-AS1* may act as a ceRNA to regulate the Rho/Rac pathway; therefore, we employed bioinformatics methods to predict potential miRNAs regulated by *AFAP1-AS1*. miR-423-5p was the only miRNA predicted by all four tools used for these analyses and had the lowest minimum free energy, suggesting that *AFAP1-AS1* is likely a direct target of miR-423-5p. Many reports have identified miR-423-5p as an oncogene in various cancers, including gastric cancer and hepatocellular carcinoma [61, 62]. In this study, we found that miR-423-5p was low-expressed in NPC and its expression was negatively correlated with the clinical TNM stages in NPC tumors. Overexpression of miR-423-5p inhibited NPC cells migration and invasion, and inhibition of miR-423-5p produced the opposite phenotype. The function of miR423-5p had an opposite effect to those of *AFAP1-AS1* on migration and invasion. Luciferase assays confirmed that miR-423-5p regulated the 3’-UTR of *AFAP1-AS1*. These results suggested that *AFAP1-AS1* may regulate downstream genes through binding of miR-423-5p, and that miR-423-5p may have a role as a tumor suppressor gene through its effects on different downstream genes in NPC.

We previously determined by the proteomic analysis that expression levels of at least 209 proteins were significantly altered after *AFAP1-AS1* knockdown in NPC cells. Among these, the expression levels of many proteins involved in regulation of cytoskeletal dynamics were confirmed as significantly altered using liquid chromatography-tandem mass spectrometry (LC-MS/MS) after *AFAP1-AS1* knockdown, including those in the small GTPase Rho/Rac signaling pathway: *RAB10, RAB11A, RAC2, PFN1, RHOA, RAC1, RHOC, LASP1*, and *RAB11B* [44]. The Rho/Rac signaling pathway is a well-established regulator of the actin cytoskeleton, as highlighted by the RAC1 dependence of membrane ruffling and RHOA-induced stress-fiber formation [63]. In this study, we focused on the role of *AFAP1-AS1* as a noncoding RNA in regulation of the expression of these proteins. We investigated whether *AFAP1-AS1* acted as a ceRNA through miR-423-5p to regulate the Rho/Rac pathway. miR-423-5p inhibited the expression of *RAC1*, *RHOC*, *LASP1*, and *RAB11B* and increased that of other molecules, including *RAB10*, *RAB11A*, *RAC2*, *PFN1*, and *RHOA*, consistently with the results after *AFAP1-AS1* inhibition or overexpression [26]. These results implied that *AFAP1-AS1* regulated the Rho/Rac signaling pathway through miR-423-5p. As a microRNA, miR-423-5p may directly regulate expression or translation of its target genes; therefore, we first examined whether some molecules including *RAC1*, *RAB11B*, *RHOC*, and *LASP1* in the Rho/Rac pathway were direct target genes of miR-423-5p, since their expression levels were reduced by the specific miR-423-5p mimics. Bioinformatics analysis predicted the *RAB11B* and *LASP1* genes as targets of miR-423-5p. Further experiments confirmed that miR-423-5p directly targeted these two genes and that *AFAP1-AS1* acted as a ceRNA of *RAB11B* and *LASP1*, regulating their expression by competitively binding miR-423-5p, thus affecting the migration and invasion of NPC. As we know, *LASP1* is an actin-binding phosphoprotein that is overexpressed in several cancers [64–66] and it is a cAMP- and cGMP-dependent signaling protein which binds to the actin cytoskeleton at extensions of the cell membrane [67, 68]. *RAB11B* belongs to the Rab family and regulates exocytotic and endocytotic pathways [69]. Our experiments confirmed the hypothesis that *AFAP1-AS1* acts as a ceRNA to regulate the expression of key genes, such as *LASP1* and *RAB11B*, by competitively binding miR-423-5p, resulting in dysregulation of the Rho/Rac1 signaling pathway and migration and invasion of human NPC cells [70–72].

The expression of *RAB10*, *RAB11A*, *RAC2*, *PFN1*, and *RHOA* were upregulated in 5-8F and HNE2 cells transfected with miR-423-5p mimics, which is consistent with the results of experiments using si*AFAP1-AS1* and suggests that *AFAP1-AS1* and miR-423-5p are tightly associated with the Rho/Rac signaling pathway. miR-423-5p may also indirectly regulate the expression of these proteins through its effects on other molecules. To identify other direct targets of miR-423-5p, we screened genes whose expression levels were highly correlated with those of *AFAP1-AS1* in NPC tissues based on the results of previous microarray analyses and the ceRNA theory. *FOSL2*, a member of the Fos family [73], was analyzed using various prediction tools and found to contain several potential miR-423-5p binding sites in its 3′-UTR, while the expression of *AFAP1-AS1* and *FOSL2* was significantly correlated in NPC GEO datasets [53]. *FOSL2* is a member of AP-1 transcription factor complexes. FOS and Jun protein family members can form homo- or heterodimers and bind to the promoters of specific genes to promote or inhibit their transcription in combination with other transcription and regulatory factors. In this study, we found that FOSL2 could regulate the *LASP1* transcription by binding its promoter, suggesting that *AFAP1-AS1* acts as a ceRNA in regulation of *FOSL2* and the AP-1 transcription factor activation, thereby affecting the expression of small GTPase molecules. Whether *AFAP1-AS1* also regulates the expression of other downstream genes through *FOSL2* is worthy of further investigation.

In this study, we confirmed that *AFAP1-AS1* functions as a ceRNA by adsorption of miR-423-5p and verified that *LASP1*, *RAB11B*, and *FOSL2* are downstream target genes of miR-423-5p (Additional file 2: Figure S5). However, miRNAs can target multiple genes, and there may be other protein-encoding genes regulated by miR-423-5p among the 209 proteins regulated by *AFAP1-AS1* in NPC cells using our previous proteomic approach; this is also worthy of further investigation.

## Conclusions

*AFAP1-AS1* may regulate a complex and sophisticated gene network through miR-423-5p as a ceRNA. Identification of the ceRNA network involving *AFAP1-AS1* may contribute to a better understanding of NPC carcinogenesis and tumor progression. Our data also suggest that targeting *AFAP1-AS1* and/or miR-423-5p have potential as a new therapeutic strategy and that these molecules are potential biomarkers in NPC.

## Additional files

Additional file 1: **Table S1.** Primers used for PCR and sequences of synthetic oligonucleotides. (PDF 8 kb)

Additional file 2: **Figure S1.** Relative miR-423-5p expression levels as determined from GEO datasets. a. miR-423-5p was downregulated in NPC biopsies (T; *n* = 62) when compared with nontumor NPE tissues (N; *n* = 6) in the GSE73460 miRNA array, *p* = 0.0008. b. miR-423-5p expression was tightly associated with the TNM stage of NPC tumors in the GSE32906 miRNA array. N, *n* = 6; I-II, *n* = 4; III, *n* = 4; IV, *n* = 4; N vs IV, *p* = 0.02; N vs III, *p* = 0.04. c. Kaplan-Meier curve analysis of overall survival (OS) and relapse-free survival (RFS) of patients with low and high miR-423-5p expression using data from the GSE70970 dataset. HR, hazard ratio. **Figure S2.** Expression of some Rho/Rac proteins, such as *RAB10*, *RAB11A*, *RAC2*, *PFN1*, *RHOA*, *RAC1*, *RHOC*, *LASP1* and *RAB11B*, was examined in 5-8F and HNE2 cells after overexpression of *AFAP1-AS1*. Figure S3. miR-423-5p could not target the 3′-UTR of *RAC1.* a. The schematic model showing the putative binding sites for miR-423-5p on 3′-untranslated regions (3′-UTR) of *RAC1*. b. Luciferase activities were measured in 5-8F and HNE2 cells cotransfected with miR-423-5p mimics, miR-423-5p inhibitors, or negative control, and the luciferase reporters containing the WT or MT *RAC1* 3′-UTR. Transfection of miR-423-5p mimics or inhibitors into 5-8F and HNE2 cells could not significantly reduce or increase the luciferase activity generated by the WT and the reporter vector containing the MT miR-423-5p binding site of the *RAC1* 3′-UTR reporter (pMIR-WT). **Figure S4.** Pearson correlation analysis was performed to evaluate the correlation between the expression of *FOSL2* and *AFAP1-AS1* using the GSE64634 dataset (*r* = 0.5256, *p* = 0.0365). **Figure S5.** A schematic model of *AFAP1-AS1* competitively binding miR-423-5p to upregulate *RAB11B* or *LASP1* or *FOSL2* transcription factor signaling and accelerate NPC metastasis. (PDF 199 kb)

## Abbreviations

*AFAP1-AS1*: Actin filament-associated protein 1 antisense RNA 1
ceRNA: Competing endogenous RNA
FBS: Fetal bovine serum
GEO: Gene expression omnibus
GEP: Gene expression profiling
LC-MS/MS: Liquid chromatography-tandem mass spectrometry
lncRNA: Long Noncoding RNA
miRNAs: MicroRNAs
MREs: MiRNA response elements
MT: Mutant
NC: Negative control
NPC: Nasopharyngeal carcinoma
PTEN: Phosphatase and tensin homolog
PTENP1: PTEN pseudogene 1
RT-PCR: Reverse transcription PCR
SD: Standard deviation
WT: Wild-type
