# Supplementary material for: Long noncoding RNA AFAP1-AS1 acts as a competing endogenous RNA of miR-423-5p to facilitate nasopharyngeal carcinoma metastasis through regulating the Rho/Rac pathway
Source: J Exp Clin Cancer Res. 2018 Oct 16;37:253. doi: 10.1186/s13046-018-0918-9 (PMC6191894; doi:10.1186/s13046-018-0918-9)
Supplement: Supplementary file 1 — Table S1. Primers used for PCR and sequences of synthetic oligonucleotides. (PDF 8 kb) [file 13046_2018_918_MOESM1_ESM.pdf]

Supplemental Table 1. Primers used for qRT-PCR or construction and siRNA

**Primers used for qRT-PCR**	
**Symbol**	**Sequence (5'-3')**
GAPDH_L	CAACGGATTTGGTCGTATTGG
GAPDH_R	TGACGGTGCCATGGAATTT
RAC1_L	TCCGTGCAAAGTGGTATCCT
RAC1_R	TCGCTTCGTCAAACACTGTC
RAC2_L	TACATCCCCACCGTGTTTGA
RAC2_R	TCATAAGAGGCTGGGCTGAC
RAB10_L	AGACCCCTGTAAAAGAGCCC
RAB10_R	AGTCAGCAAGCAGTCACAGA
RAB11A_L	CTACGTCATCTCAGGGCAGT
RAB11A_R	ACTGCACCTTTGGCTTGTTT
RAB11B_L	TTCAAAGTGGTGCTCATCGG
RAB11B_R	TCATAGGTCAGGTGCTTGGC
RHOA_L	CACAGTGCTCTTTTCTCCTCA
RHOA_R	AGTTTAGTCAGCTGGAGAGAAG
RHOC_L	GCAATCCGAAAGAAGCTGGT
RHOC_R	TCCACCTCAATGTCCGCAAT
LASP1_L	GAAGAAGCCCTACTGCAACG
LASP1_R	TTGATTCTCTGGAGCTCGGG
PFN1_L	CCAAGACTGACAAGACGCTAG
PFN1_R	AAGGGGTATGGGGTAATGGC
FOSL2_L	GCCAGCAGAAATTCCGGG
FOSL2_R	GGGTTGGACATGGAGGTGAT
AFAP1-AS1_L	AATGGTGGTAGGAGGGAGGA
AFAP1-AS1_R	CACACAGGGGAATGAAGAGG
**The wild type and mutant seqences for vectors construction**	
**Symbol**	**Sequence (5'-3')**
423-5p-AFAP1-AS1-WT_L	CTAGTTTTCCAGGTGTGAGCTGCTCCTCTCCCCCATCTGCCCCTCCTCAAAGAAACAA
423-5p-AFAP1-AS1-WT_R	AGCTTTGTTTCTTTGAGGAGGGGCAGATGGGGGAGAGGAGCAGCTCACACCTGGAAAA
423-5p-AFAP1-AS1-MT_L	CTAGTTTTCCAGGTGTGAGCTGCTCCTCTCCCCCACTCAAAGAAACAA
423-5p-AFAP1-AS1-MT_R	AGCTTTGTTTCTTTGAGTGGGGGAGAGGAGCAGCTCACACCTGGAAAA
423-5p-FOSL2-WT _L	CTAGTGCCTGGACCTTCCCCAGATGCTGCCAGGCAGCCCCTCCCCAAGCCTCAAAGAA
423-5p-FOSL2-WT _R	AGCTTTCTTTGAGGCTTGGGGAGGGGCTGCCTGGCAGCATCTGGGGAAGGTCCAGGCA
423-5p-FOSL2-MT_L	CTAGTGCCTGGACCTTCCCCAGATGCTGCCAGGCA CTAGGCACCCAAGCCTCAAAGAA
423-5p-FOSL2-MT_R	AGCTTTCTTTGAGGCTTGGGTGCCTAGTGCCTGGCAGCATCTGGGGAAGGTCCAGGCA
pAP1-PFN1-WT _L	CCGCGGCTGAGTCACCGCGGCTGAGTCACCGCGGCTGAGTCACA
pAP1-PFN1-WT _R	GATCTGTGACTCAGCCGCGGTGACTCAGCCGCGGTGACTCAGCCGCGGGTAC
pAP1-PFN1-MT_L	CTGCGGCCTGGGACCTGCGGCCTGGGACCTGCGGCCTGGGACCA
pAP1-PFN1-MT_R	GATCTGGTCCCAGGCCGCAGGTCCCAGGCCGCAGGTCCCAGGCCGCAGGTAC
423-5p-RAC1-WT_L	CTAGTAGAAAATGCCTGCTGTTGTAAATGTCTCAGCCCCTCGTTCTTGGTCCTGTCCA
423-5p-RAC1-WT_R	AGCTTGGACAGGACCAAGAACGAGGGGCTGAGACATTTACAACAGCAGGCATTTTCTA
423-5p-RAC1-MT_L	CTAGTAGAAAATGCCTGCTGTTGTAAATGTCTCACAAATGTGTTCTTGGTCCTGTCCA
423-5p-RAC1-MT_R	AGCTTGGACAGGACCAAGAACACATTTGTGAGACATTTACAACAGCAGGCATTTTCTA
423-5p-RAB11B-WT_L	CTAGTCCGCCCGCCCCCGCCACGGTATCCTCTGGCCCCTCCCTGCTGTCCCTCTGTGA
423-5p-RAB11B-WT_R	AGCTTCACAGAGGGACAGCAGGGAGGGGCCAGAGGATACCGTGGCGGGGGCGGGCGGA
423-5p-RAB11B-MT_L	CTAGTCCGCCCGCCCCCGCCACGGTATCCTCTGAATATGACCTGCTGTCCCTCTGTGA
423-5p-RAB11B-MT_R	AGCTTCACAGAGGGACAGCAGGTCATATTCAGAGGATACCGTGGCGGGGGCGGGCGGA
423-5p-LASP1-WT_L	CTAGTGTGGGCCTCACCTGCCCCTCTGTTCTCTCCCCTCACATCCTCCTGCCCAGCTA
423-5p-LASP1-WT_R	AGCTTAGCTGGGCAGGAGGATGTGAGGGGAGAGAACAGAGGGGCAGGTGAGGCCCACA
423-5p-LASP1-MT_L	CTAGTGTGGGCCTCACCTGCCCCTCTGTTCTCTTTAAGTCCATCCTCCTGCCCAGCTA
423-5p-LASP1-MT_R	AGCTTAGCTGGGCAGGAGGATGGACTTAAAGAGAACAGAGGGGCAGGTGAGGCCCACA

**The primers for the AFAP1-AS1 overexpression vectors**	
**Symbol**	**Sequence (5'-3')**
AFAP1-AS1_L	GCTGGCTAGCGTTTAAACTTAAGCTTCAGTGCCTCCCTCGCTCAAT
AFAP1-AS1_R	TGCAGAATTCCACCACACTGGATCCTTTGTTTGACTTTGTGTTTATTTTTAATG
**siRNAs**	
**Symbol**	**Sequence (5'-3')**
siAFAP1-AS1	CCTATCTGGTCAACACGTATT
siFOSL2	AAGACCUGGCGUGAUCAAGACCAUU
