# Supplementary material for: Long noncoding RNA AFAP1-AS1 acts as a competing endogenous RNA of miR-423-5p to facilitate nasopharyngeal carcinoma metastasis through regulating the Rho/Rac pathway
Source: J Exp Clin Cancer Res. 2018 Oct 16;37:253. doi: 10.1186/s13046-018-0918-9 (PMC6191894; doi:10.1186/s13046-018-0918-9)

Supplementary Figure S1

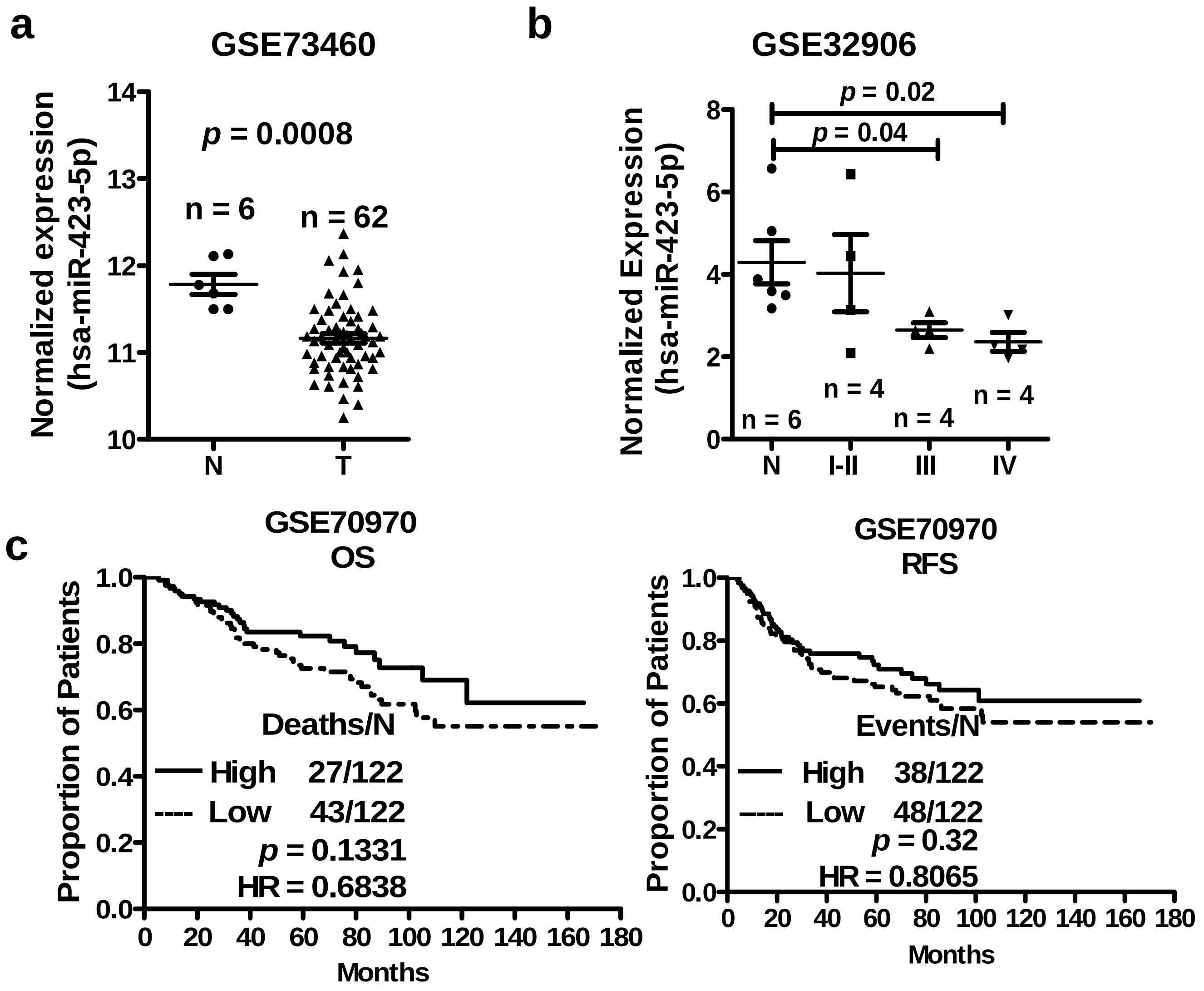

Supplementary Figure S2

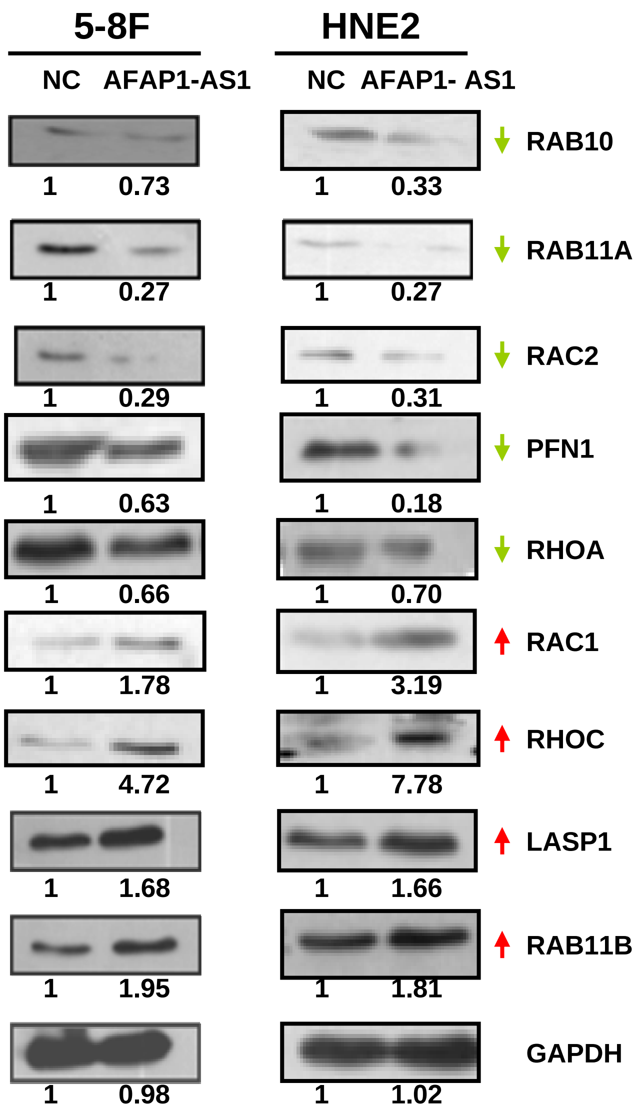

Supplementary Figure S3

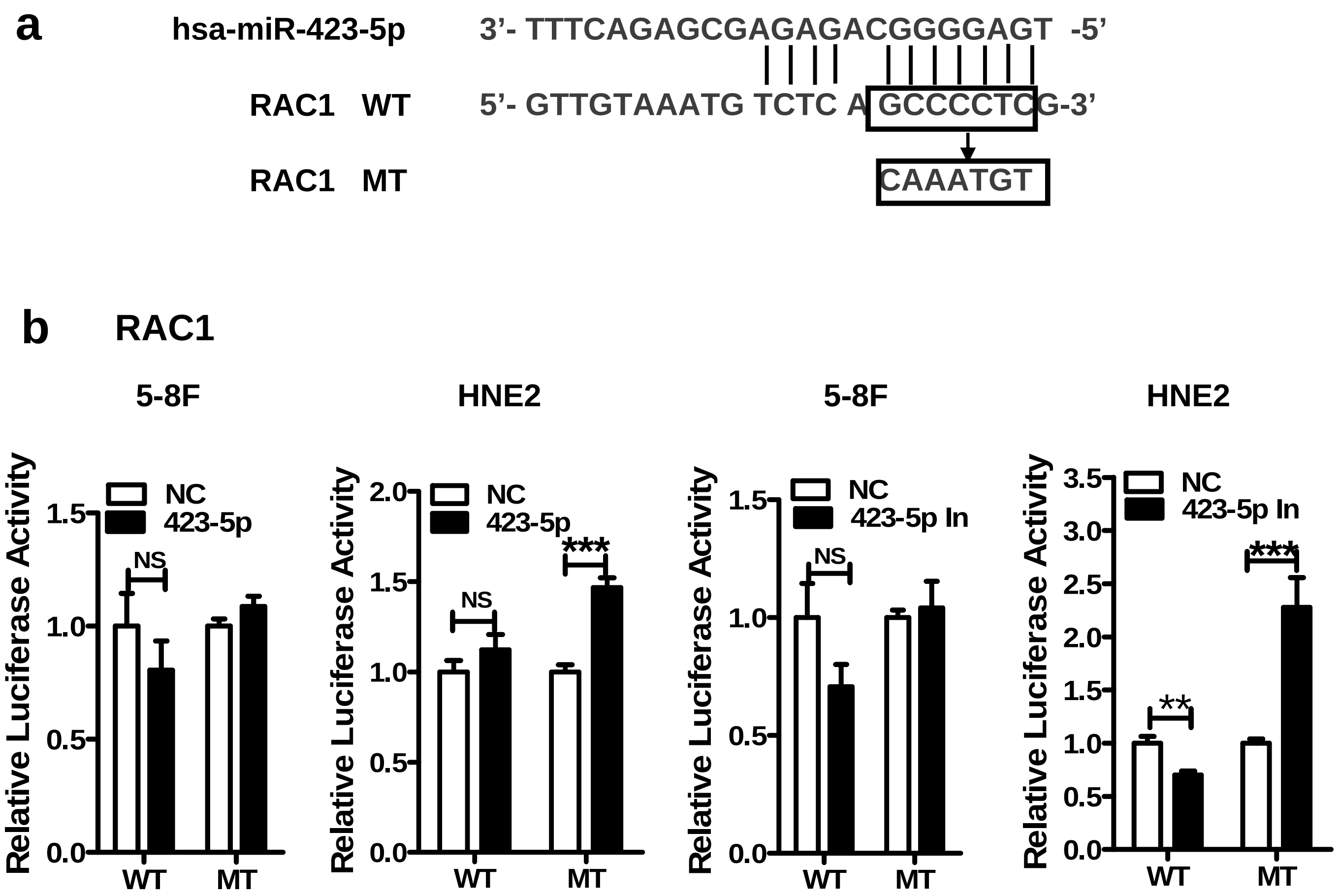

Supplementary Figure S4

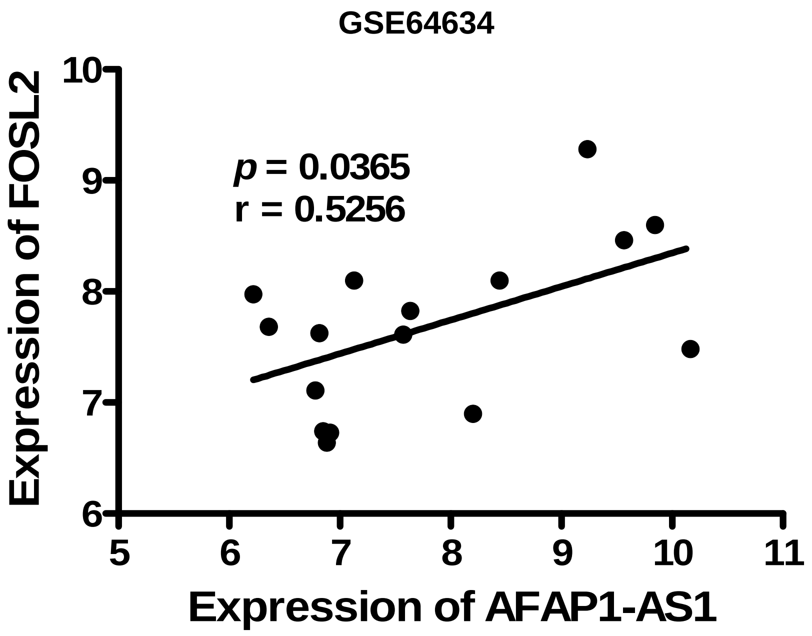

Supplementary Figure S5

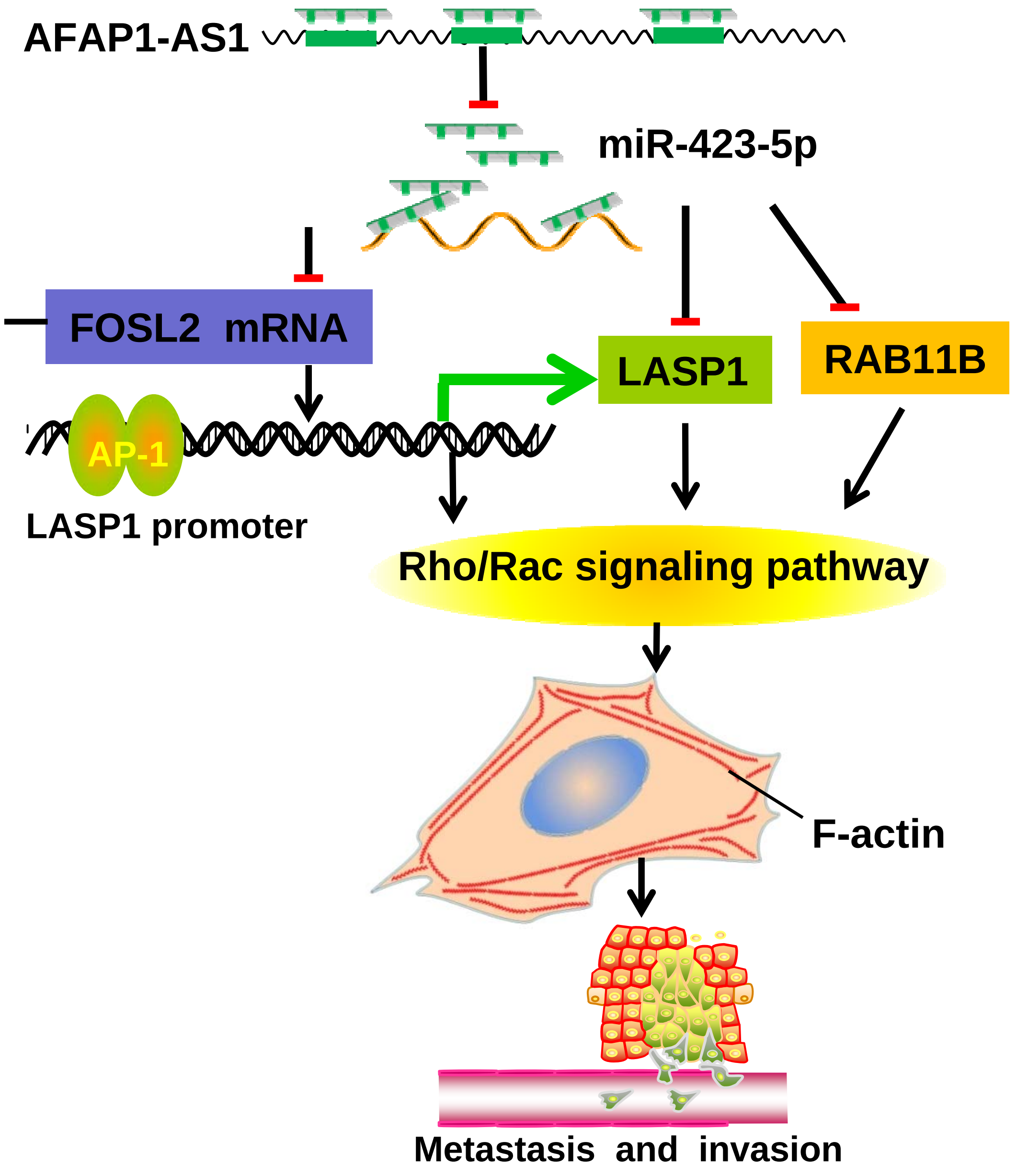

Supplement: Supplementary file 2 — Figure S1. Relative miR-423-5p expression levels as determined from GEO datasets. a. miR-423-5p was downregulated in NPC biopsies (T; n = 62) when compared with nontumor NPE tissues (N; n = 6) in the GSE73460 miRNA array, p = 0.0008. b. miR-423-5p expression was tightly associated with the TNM stage of NPC tumors in the GSE32906 miRNA array. N, n = 6; I-II, n = 4; III, n = 4; IV, n = 4; N vs IV, p = 0.02; N vs III, p = 0.04. c. Kaplan-Meier curve analysis of overall survival (OS) and relapse-free survival (RFS) of patients with low and high miR-423-5p expression using data from the GSE70970 dataset. HR, hazard ratio. Figure S2. Expression of some Rho/Rac proteins, such as RAB10, RAB11A, RAC2, PFN1, RHOA, RAC1, RHOC, LASP1 and RAB11B, was examined in 5-8F and HNE2 cells after overexpression of AFAP1-AS1. Figure S3. miR-423-5p could not target the 3′-UTR of RAC1. a. The schematic model showing the putative binding sites for miR-423-5p on 3′-untranslated regions (3′-UTR) of RAC1. b. Luciferase activities were measured in 5-8F and HNE2 cells cotransfected with miR-423-5p mimics, miR-423-5p inhibitors, or negative control, and the luciferase reporters containing the WT or MT RAC1 3′-UTR. Transfection of miR-423-5p mimics or inhibitors into 5-8F and HNE2 cells could not significantly reduce or increase the luciferase activity generated by the WT and the reporter vector containing the MT miR-423-5p binding site of the RAC1 3′-UTR reporter (pMIR-WT). Figure S4. Pearson correlation analysis was performed to evaluate the correlation between the expression of FOSL2 and AFAP1-AS1 using the GSE64634 dataset (r = 0.5256, p = 0.0365). Figure S5. A schematic model of AFAP1-AS1 competitively binding miR-423-5p to upregulate RAB11B or LASP1 or FOSL2 transcription factor signaling and accelerate NPC metastasis. (PDF 199 kb) [file 13046_2018_918_MOESM2_ESM.pdf]
